# Simulation-Based Design of an Electrically Tunable Beam-Steering Metasurface Driven by a Triboelectric Nanogenerator

**DOI:** 10.3390/mi16080948

**Published:** 2025-08-19

**Authors:** Penghui Luo, Longlong Zhang, Shuaixing Wang, Zhiyuan Zhu

**Affiliations:** 1 Jiangxi Provincial Key Laboratory of Lightweight Composite Materials (Nanchang Hangkong University), Nanchang Hangkong University, Nanchang 330063, China; lph15025787847@email.swu.edu.cn (P.L.); wsxxpg@163.com (S.W.); 2 College of Electronic and Information Engineering, Southwest University, Chongqing 400715, China; a020100@email.swu.edu.cn; 3 Hubei Key Laboratory of Transportation Internet of Things, Wuhan University of Technology, Wuhan 430070, China

**Keywords:** triboelectric nanogenerator (TENG), reconfigurable metasurface, beam steering, electromagnetic wave control

## Abstract

This study presents a simulation-based feasibility analysis of a beam steering metasurface, theoretically driven by mechanical energy harvested from human motion via a triboelectric nanogenerator (TENG). In the proposed model, the TENG converts biomechanical motion into alternating current (AC), which is rectified into direct current (DC) to bias varactor diodes integrated into each metasurface unit cell. These bias voltages are numerically applied to dynamically modulate the local reflection phase, enabling beam steering without external power. Full-wave electromagnetic simulations were conducted to confirm the feasibility of beam manipulation under TENG-generated voltage levels. The proposed simulation-driven design offers a promising framework for battery-free, adaptive electromagnetic control with potential applications in wearable electronics, intelligent sensing, and energy-autonomous radar systems.

## 1. Introduction

Amid the accelerating evolution of intelligent sensing [1], wearable electronics [2,3], and passive radar technologies [4], the pursuit of self-sustaining electromagnetic functional devices [5]—such as reconfigurable metasurface [6], antenna arrays [7], and wireless sensor nodes [8]—has emerged as a focal point in the advancement of next-generation adaptive systems [9]. These devices are capable of autonomous operation by harvesting ambient mechanical energy, offering strong potential for integration into portable, wearable, and embedded platforms. Conventional beam steering systems typically rely on external power supplies and complex control circuitry, which not only increase the system’s size and power consumption but also limit their integration in portable and embedded applications [10,11,12]. Therefore, realizing beam manipulation systems that are battery-free, structurally compact, and capable of adaptive control has become a key research direction in the field of intelligent electromagnetic materials and systems [13,14,15,16].

Triboelectric nanogenerator (TENG) is an emerging micro/nano energy harvester that converts mechanical energy into electrical output through the coupling of contact electrification and electrostatic induction [17,18,19]. Due to their simple structure, high voltage output, and high sensitivity, TENG is particularly suitable for harvesting low-frequency and irregular mechanical energy from human motion [20,21,22,23]. In recent years, they have been widely applied in wearable electronics, environmental energy harvesting, and low-power sensors [24,25,26]. However, the direct application of TENG output to dynamically control electromagnetic metasurfaces—especially for manipulating and deflecting reflected beams—still faces several challenges, including unclear operational mechanisms and low system integration [27,28,29].

This study proposes an electrically tunable metasurface beam steering system driven by a TENG. The system utilizes an integrated TENG to harvest mechanical energy, converting it into electrical energy for biasing the varactor diodes without external power. The alternating current (AC) output from the TENG is rectified into direct current (DC) and applied to varactor-integrated metasurface unit cells. The applied voltage modulates the reflection phase of each unit, enabling beam steering. Full-wave simulation results at a microwave frequency range of 4.0–4.4 GHz demonstrate that a beam steering angle of approximately 20–40° can be achieved by tuning the TENG’s output voltage. These results confirm the feasibility of using TENG to enable beam steering without any external power source.

The key innovation of this work lies in the integration of mechanically driven energy harvesting and dynamic metasurface control technologies. A novel, self-sustained beam control architecture is developed, featuring high adaptability, low power consumption, and excellent integrability. The remainder of this paper is organized as follows: Section 2 discusses the metasurface theory based on TENG control; Section 3 presents the energy generation mechanism and simulation of TENG output; Section 4 introduces the rectification circuit and power conversion process; Section 5 analyzes the principle and simulation of beam steering control; Section 6 discusses the results; and finally, Section 7 summarizes the conclusions and future perspectives.

## 2. TENG-Driven Metasurface Theory

**Figure 1:**
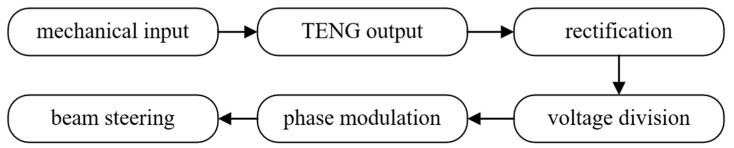
Flowchart of triboelectric nanogenerator control of metasurface.

To achieve TENG-driven and tunable electromagnetic beam manipulation, we propose an active metasurface powered by a triboelectric nanogenerator, which functions as a micro-/nano-scale energy harvester. TENG converts mechanical motion—such as human gestures, structural vibrations, or environmental disturbances—into alternating current electrical output. This output is subsequently rectified into a direct current control signal to bias varactor diodes embedded in each metasurface unit cell, thereby enabling dynamic phase modulation and ultimately achieving beam steering without an external power supply; its flowchart is shown in Figure 1.

**Figure 2:**
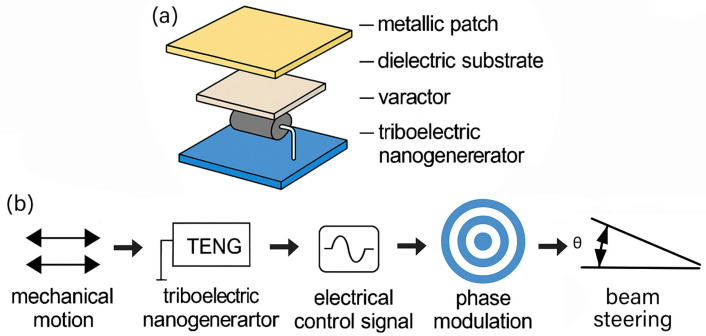
Schematic diagram of TENG-driven beam deflection system. (**a**) Unit structure. (**b**) Operating principle.

The proposed metasurface unit is an active element powered by a triboelectric nanogenerator as illustrated in Figure 2a, which acts as a micro/nanoscale mechanical energy harvester. The unit cell consists of a metallic patch, a dielectric substrate, a varactor diode, and an integrated TENG module. The complete operating principle is schematically depicted in Figure 2b. Mechanical motion first activates the TENG, which generates an AC signal. After rectification and filtering, this signal becomes a DC bias applied to the varactor diode. The resulting change in capacitance leads to a modulation of the reflected wave’s phase from the metasurface. This mechanism enables dynamic control of the beam direction—known as beam steering. This approach eliminates the need for external wiring or battery-powered control circuitry, offering significant advantages in terms of integration, scalability, and deployment in remote or constrained environments.

## 3. Electrical Output Characteristics of TENG

### 3.1. Fundamental Working Principle of TENG

The TENG operates based on the principle of contact electrification and electrostatic induction, wherein two materials with differing electron affinity (such as a metal and a polymer) generate surface charges upon repeated contact and separation. When the two surfaces are brought into frictional contact, electrons are transferred from one material to the other due to their intrinsic difference in electron affinity, creating a dipole layer at the interface. Upon separation, this charge imbalance induces a potential difference, which drives electron flow through an external circuit, thereby generating electricity.

Depending on the selected material properties, the triboelectric charges can either flow immediately through a conductor to power external devices or remain accumulated on insulating or weakly conductive surfaces. In essence, triboelectric energy harvesting relies on the transfer and manipulation of surface charges between contact materials.

The working modes of TENG are typically categorized into four types: vertical contact–separation mode, lateral sliding mode, single-electrode mode, and freestanding triboelectric-layer mode. Compared with other working modes, the vertical contact–separation mode adopted in this work offers a simpler structure, better compatibility with perpendicular mechanical motion (e.g., human pressing), and more stable electrical output. These features make it particularly suitable for integration with planar metasurface structures and wearable platforms. In this work, a vertical contact–separation mode is adopted to harvest biomechanical energy—primarily generated from human body motion—by periodically pressing a pair of triboelectric materials to produce a transient electrical signal, which is then directed into an external circuit to power or control subsequent components.

**Figure 3:**
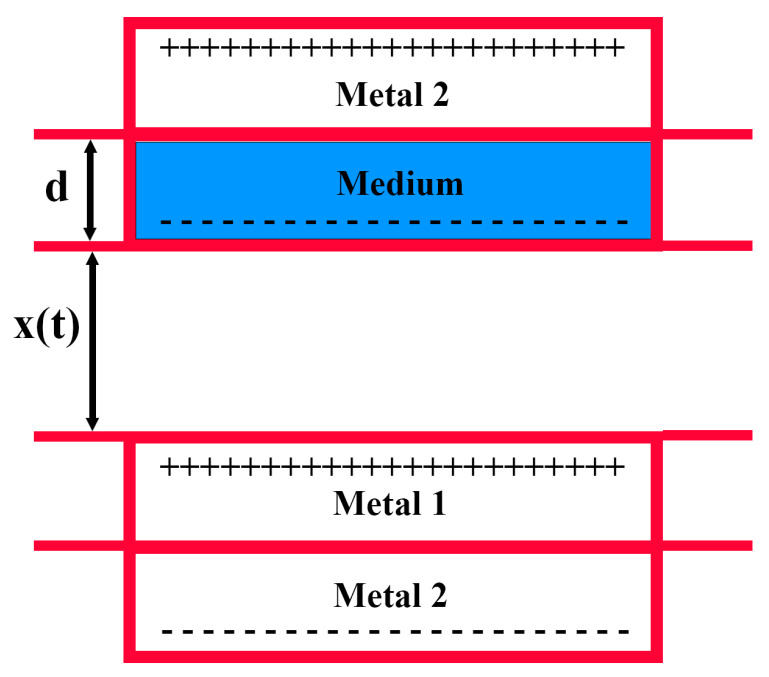
Theoretical model of vertical contact–separation mode TENG.

Mechanistically, a vertical contact–separation TENG can be modeled as a variable capacitor. As shown in Figure 3, the two triboelectric materials act as the capacitor’s plates. Upon contact, charge transfer occurs between the two materials. When separated, a distance-dependent potential difference is generated across the electrodes, driving charge flow. With repeated contact and release cycles, equal amounts of opposite charges accumulate on the surfaces of the triboelectric layers. The metal electrode on each side, influenced by the surface-bound charges, becomes induced with image charges of equal magnitude but opposite sign, thus establishing a periodic electrical output.

According to fundamental electrostatic theory, the potential difference between two electrodes can be expressed as

(1) $$V=-\frac{Q}{C^{\prime }\left(x\right)}+V_{c}\left(x\right)$$

where *C′* (*x*) represents the position-dependent equivalent capacitance between the electrodes, and *V_c_* (*x*) denotes the corresponding potential difference across the plates.

(2) $$V_{c}\left(x\right)=\frac{Q}{C\left(x\right)}=\sigma S/\frac{\varepsilon_{0}S}{x\left(t\right)}=\frac{\sigma x(t)}{\varepsilon_{0}}$$

where *σ* is the surface charge density on the triboelectric material.

Combining Equations (1) and (2), the potential difference between the electrodes can be further expressed as

(3) $$V=-\frac{Q}{\varepsilon_{0}S}\left(d+x\left(t\right)\right)+\frac{\sigma x(t)}{\varepsilon_{0}}$$

where *S* denotes the effective contact area of the triboelectric layers, and d represents the thickness of the dielectric material. When *d* is much smaller than the separation distance between the triboelectric layers, it can be reasonably neglected.

In the experimental setup for the triboelectric unit, an arbitrary load resistance *R* can be connected. According to Ohm’s law, the instantaneous current flowing through the circuit can then be expressed as

(4) $$R\frac{dQ}{dt}=-\frac{Q}{\varepsilon_{0}S}\left(d+x\left(t\right)\right)+\frac{\sigma x(t)}{\varepsilon_{0}}$$

### 3.2. Fabrication and Working Principle of TENG

**Figure 4:**
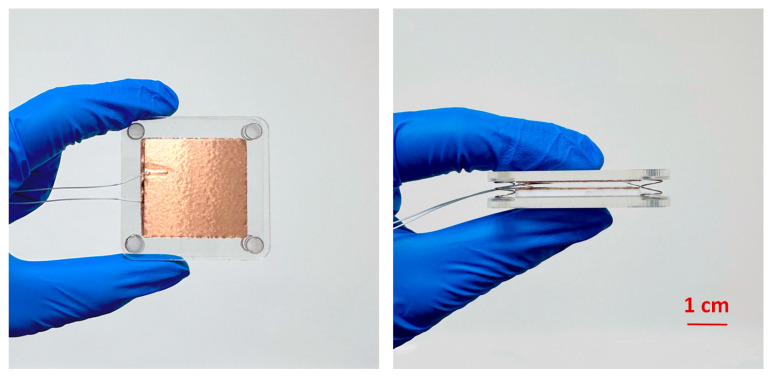
The fabrication of TENG.

This study designs a TENG that adopts the contact–separation mode of operation, as shown in Figure 4. All of the experimental instruments and materials used in this chapter are shown in Table 1. To implement the TENG, polydimethylsiloxane (PDMS, 0.1 mm thick) and aluminum foil (0.05 mm thick) are employed as the triboelectric pair. Copper foil is laminated on the back side of both materials to collect and transfer charge. The entire TENG structure primarily consists of two transparent acrylic substrates serving as the top and bottom plates. These square acrylic plates (50 mm in length and 3 mm in thickness) were fabricated via laser cutting, offering lightweight properties and excellent impact resistance. For optimal contact performance, energy output, and spatial efficiency, the TENG module is fabricated into a 35 mm × 35 mm rectangular structure, ensuring sufficient frictional interaction under periodic mechanical excitation.

**Figure 5:**
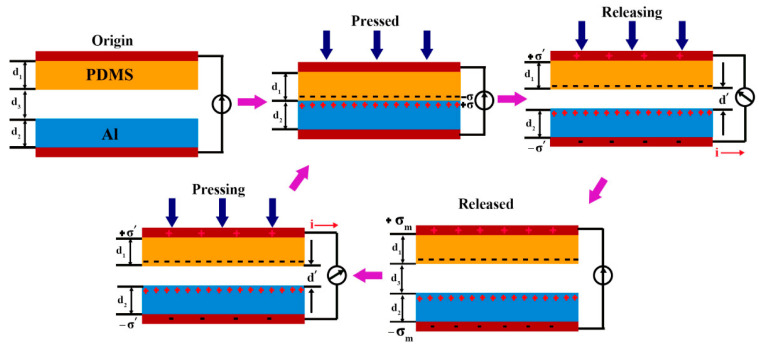
The operating mechanism of TENG.

Under static conditions, the triboelectric layers—polydimethylsiloxane (PDMS) and aluminum (Al) foil—remain electrically neutral due to electrostatic equilibrium. However, repeated contact and separation between these two materials result in the accumulation of opposite charges on their respective surfaces. When fully in contact, these equal but opposite charges neutralize each other, leading to no observable potential difference. Once the materials are separated, a potential difference is established between the PDMS and Al foil, and, if connected via an external circuit, electrons flow from the PDMS to the Al foil. Conversely, during compression when the materials approach each other, this potential difference decreases, causing electrons to flow in the opposite direction—from Al foil to PDMS—until full contact is reestablished and neutrality is restored. The complete mechanical-to-electrical energy conversion mechanism of the designed TENG is illustrated in Figure 5.

**Figure 6:**
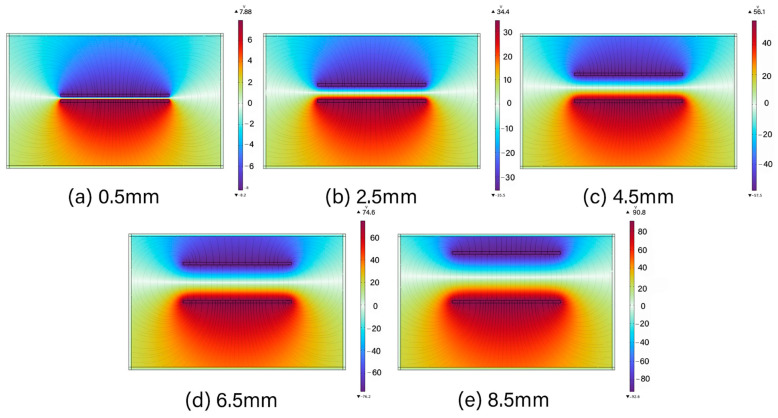
Interlayer potential variation with gap distance in TENG.

To further validate the proposed working mechanism, electrostatic simulations were performed using COMSOL Multiphysics 6.0. The finite element analysis (FEA) was employed to model the electrostatic interactions between the Al foil and PDMS layers. The simulation results as presented in Figure 6, confirm the presence of charge redistribution and potential variation in response to contact–separation motion, thereby supporting the theoretical operation of the TENG. This indicates that as the vertical gap between the upper and lower triboelectric layers increases, a larger potential difference is established between the separated surfaces, which in turn drives electron flow in the external circuit.

### 3.3. Performance Characterization of TENG

**Figure 7:**
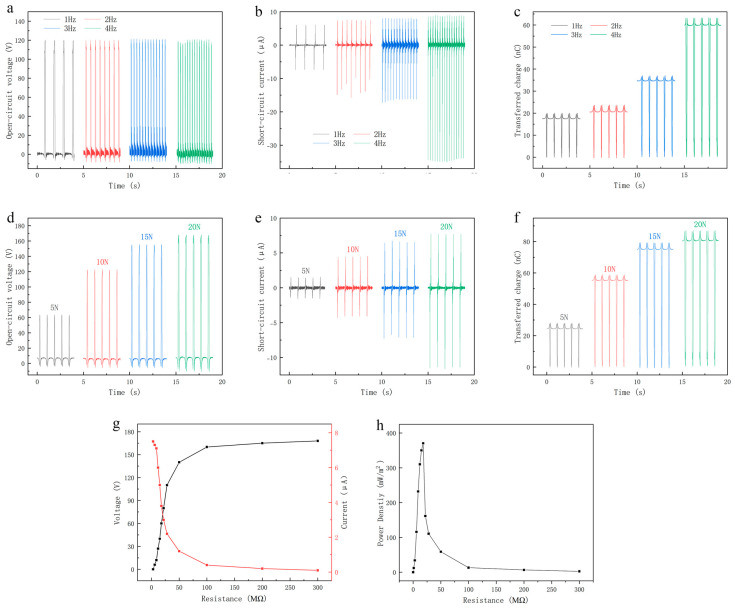
Output performance of TENG. (**a**) Open-circuit voltage at different contact–separation frequencies. (**b**) Short-circuit current at different contact–separation frequencies. (**c**) Transferred charge at different contact–separation frequencies. (**d**) Open-circuit voltage under different applied stresses. (**e**) Short-circuit current under different applied stresses. (**f**) Transferred charge under different applied stresses. (**g**) Measured output voltage and current on different external loading resistances. (**h**) Power density on different external loading resistances.

Considering the influence of large frequency fluctuations under motion conditions, in order to further study the output characteristics of the TENG, it is necessary to explore the relationship between the output performance of the device and the frequency under normal motion frequencies. As shown in Figure 7, when the contact–separation distance is set to 40 mm, the TENG operates at various motion frequencies (1 Hz, 2 Hz, 3 Hz, and 4 Hz). The results show that the open-circuit voltage remains relatively stable at approximately 120 V across different frequencies in Figure 7a. In contrast, the short-circuit current increases with rising frequency, from about 6 μA at 1 Hz to around 8 μA at 4 Hz in Figure 7b. This indicates that higher frequencies lead to greater short-circuit currents, while having minimal influence on the open-circuit voltage. This behavior can be attributed to the fact that, under otherwise constant experimental conditions, the amount of transferred charge remains stable, while the number of contact–separation cycles increases with frequency. Figure 7c shows the increasing transferred charges as the frequency rises. As a result, the charge transfer rate is enhanced, yielding higher current output.

Given that the TENG is sensitive to external mechanical stress, the relationship between applied pressure and its output performance was further explored. A force gauge was used to apply different normal pressures (5 N, 10 N, 15 N, and 20 N) to the device surface, with a constant contact–separation frequency of 1 Hz and a fixed distance of 40 mm. Figure 7d illustrates the open-circuit voltage under varying applied forces. At 5 N, the voltage output is approximately 63 V, increasing steadily with greater applied force, and reaching a maximum of about 168 V at 20 N. In addition, the short-circuit current under different pressure conditions was measured, as shown in Figure 7e. The current rises from 1.3 μA at 5 N to 7.5 μA at 20 N. These results are consistent with expectations and confirm the TENG’s high sensitivity to external stress. Figure 7f shows the increasing transferred charges as the external force rises. The clear trend observed in the data provides a solid foundation for evaluating the reliability and stability of the TENG under varying pressure conditions. In Figure 7g, we show the output performance of the TENG with different external resistance. With the increasing resistance, the instantaneous power increased steadily at first and then tended to decrease when it reached the peak value. The corresponding power density is 370.5 mW/m^2^ with external loading resistance of 20 MΩ in Figure 7h, demonstrating the good triboelectric characteristic.

Compared with typical low-frequency piezoelectric and electromagnetic (EM) energy harvesters, the TENG-driven metasurface exhibits distinct advantages. Low frequency piezoelectric harvesters generally produce 1–50 mW/m^2^, while EM harvesters generate 10–100 mW/m^2^ under similar motion conditions. In contrast, this TENG delivers higher power density up to 370.5 mW/m^2^ in the low-frequency regime, making it particularly suitable for human-motion or environmental-vibration applications. Moreover, the TENG output is highly tunable: the short-circuit current increases with both frequency and applied force, while the open-circuit voltage remains relatively stable, offering a wide dynamic range that surpasses the limited strain range of piezoelectric materials and the narrow frequency response of EM harvesters. This combination of high power density and flexible tunability makes the TENG-driven metasurface especially advantageous for integration with mechanically modulated beam-steering metasurfaces.

**Figure 8:**
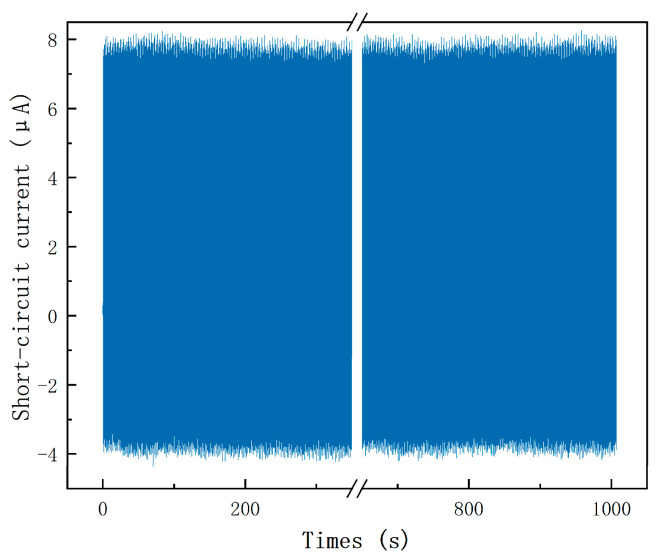
The mechanical stability of TENG in 1000 working cycles.

The stability and durability of the TENG device in this study were rigorously evaluated. A simulated test environment was established using a linear motor system, where an external force of 30 N was periodically applied to the fabricated TENG at a frequency of 1 Hz. This setup was designed to mimic real-world operating conditions over extended periods and to assess whether the device could maintain stable output performance under repeated mechanical cycling. The objective of this cyclic durability test was to verify the long-term operational reliability of the TENG. After 1000 working cycles, the short-circuit current output was recorded, as shown in Figure 8. It can be observed that the initial short-circuit current output was approximately 8 μA when the linear motor first applied stress. Remarkably, after 1000 consecutive cycles, the current remained consistently around 8 μA, showing no significant degradation.

Beyond periodic operation, the TENG can also be driven by irregular mechanical inputs, such as human motion. As an intermittent energy harvester, the TENG collects charges during each mechanical activation, regardless of input periodicity, and stores the harvested energy for subsequent delivery to the metasurface. While non-periodic excitation may lead to fluctuations in instantaneous output, the charging–discharging nature of the TENG ensures reliable powering capability once sufficient charge has been accumulated. These results confirm that the device maintains stable performance under both regular and irregular inputs, highlighting its durability and adaptability for practical beam-steering applications.

## 4. Energy Conversion Based on Rectifier Circuit

**Figure 9:**
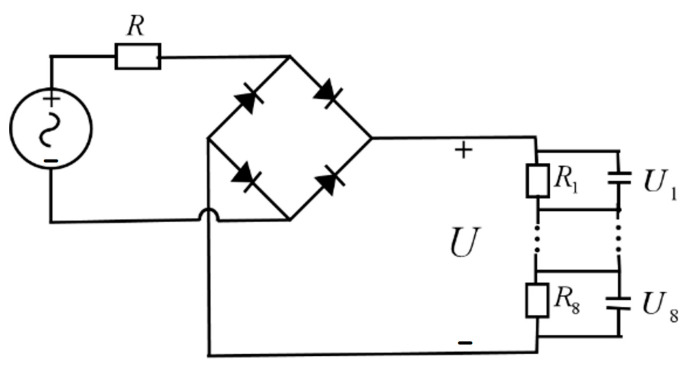
The circuit simulation diagram.

Based on the results from the previous Section, a pressing motion with a frequency of 4 Hz at 20 N was selected for the TENG. This motion generates an alternating voltage with a peak magnitude of 168 V. However, due to the inherently high internal resistance of the triboelectric materials and the fact that AC voltage cannot be directly applied to the metasurface, a rectifier circuit is required to convert the AC output into DC. To obtain the actual output voltage, a simulation was conducted in Multisim using the circuit shown in Figure 9. The rectifier uses high-voltage, low-leakage small-signal silicon diodes to minimize loading of the high-impedance TENG source. At 4 Hz, the leakage current is much smaller than the TENG’s source-limited current, so their impact on the DC output is minor. For additional margin, two diodes in series per bridge leg may be used with a further reduction in effective capacitance. Then the internal resistance of TENG is set to R = 20 MΩ, and several voltage divider resistors are included in the external circuit, which are intended to provide power to the metasurface.

In the Multisim simulation, the resistance values of the resistors in the DC output section of the rectifier bridge vary depending on their intended application. To maximize the energy output, equivalent resistors of 20 MΩ are used to replace these resistors. However, since the beam-steering metasurface design requires voltage control, it will be adjusted according to the phase requirements. The specific configuration is discussed in the following Section.

## 5. Electrically Tunable Beam-Steering Metasurface

### 5.1. Principle of Beam-Steering

**Figure 10:**
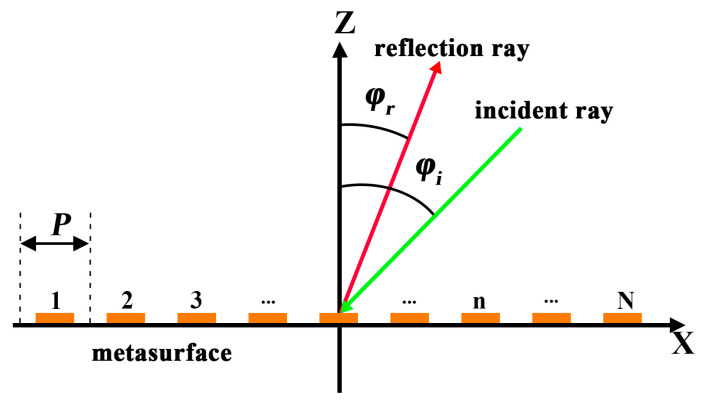
The beam steering schematic diagram.

As illustrated in Figure 10, the metasurface is positioned in the xoy plane, demonstrating the effect of beam steering. For ease of analysis, the active metasurface units are sequentially numbered from 1 to N, with ***P*** denoting the periodicity of the metasurface units along the x-axis. A plane electromagnetic wave, incident in the xoz plane at an angle ***φᵢ***, impinges on the metasurface and is reflected at an angle ***φᵣ***.

According to the generalized Snell’s law of reflection, when a plane electromagnetic wave is incident on the metasurface at an angle ***φᵢ***, the reflected wave can be redirected to a desired angle ***φᵣ***, by imposing a linear phase gradient across the surface. The reflection phase ***Φₙ*** of the ***n***th unit cell must satisfy as

(5) $$\Phi_{n}=\Phi_{n-1}-k_{0}P(sin\varphi_{r}-sin\varphi_{i})$$

where ***k*****_0_** is the free-space wavenumber and ***P*** is the period of adjacent unit cells. This relation stems from the momentum conservation parallel to the metasurface, and the imposed phase gradient effectively alters the direction of the reflected wavefront. Intuitively, the metasurface acts as a spatial phase modulator; by tailoring the reflection phase of each unit cell, the entire surface introduces a controlled phase shift across the aperture, enabling the anomalous reflection of electromagnetic waves at non-specular angles.

### 5.2. Electrically Tunable Beam-Steering Metasurface Unit Design Simulation

**Figure 11:**
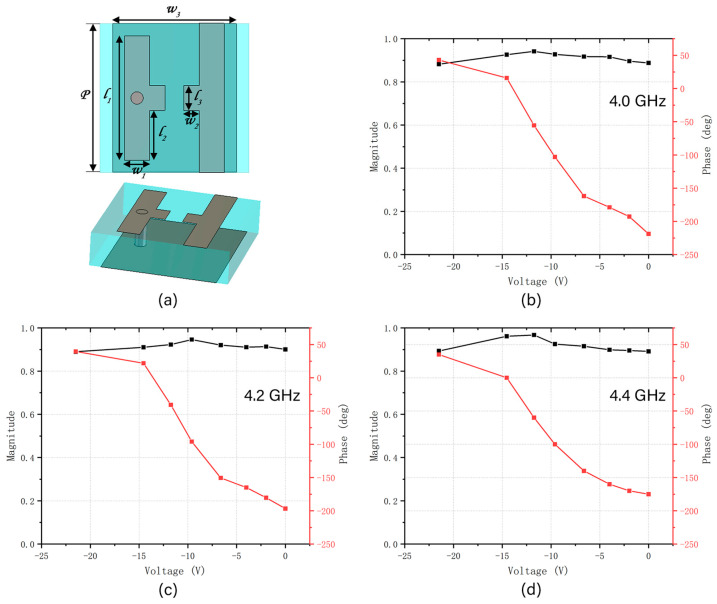
Electrically tunable metasurface unit and simulation results. (**a**) Schematic of the metasurface unit structure. (**b**) Reflection coefficient magnitude and phase as functions of voltage at 4.0 GHz. (**c**) Reflection coefficient magnitude and phase as functions of voltage at 4.2 GHz. (**d**) Reflection coefficient magnitude and phase as functions of voltage at 4.4 GHz.

To comprehensively evaluate the tunable reflection characteristics of the metasurface unit, full-wave electromagnetic simulations were conducted using CST Studio Suite 2023.04 under multiple operating conditions. The unit cell was modeled with periodic boundary conditions along the x- and y-directions. A normally incident plane wave propagated along the z-direction, while Floquet ports were employed to excite the structure and extract the reflected wave information. Figure 11a shows the unit structure of the metasurface, which consists of a structural layer, a feed layer, and a substrate. The structural layer is made up of two parallel metal plates, with a varactor diode integrated in between. The light blue area represents the TB-73 substrate, located between the structural and feed layers, with a dielectric constant of 4.3, a loss tangent of 0.001, and a thickness of 3 mm. The feed layer has a through hole that connects with the structural layer. The gray area represents the feed layer, which enables flexible arrangement variations. All metal structures are made of copper with a thickness of 0.017 mm. The individual size of the unit is 12 × 12 mm^2^. The dimensions of the unit structure are shown in Table 2.

The metasurface unit contains a varactor diode, and the phase of the reflected planar electromagnetic wave changes with the applied bias voltage, enabling the design of a phase-tunable metasurface. To simulate the tuning effect of the varactor diode, a lumped element boundary was inserted between the patterned patch and ground to represent the equivalent capacitance under different bias voltages. In this design, the SMV1430-040LF varactor diode (Skyworks Solutions, Inc., Woburn, MA, USA) was selected. Its capacitance varies non-linearly from 1.284 pF to 0.367 pF as the applied bias voltage increases from 0 V to 23 V. The metasurface unit loaded with the varactor diode was simulated with CST Studio Suite 2023.04. When different bias voltages are applied, the metasurface unit exhibits varying magnitudes and reflection phases in response to normally incident planar electromagnetic waves.

To evaluate the performance at different frequencies, simulations were conducted at 4.0 GHz, 4.2 GHz, and 4.4 GHz, as shown in Figure 11b–d. Three frequency scenarios show a continuous and monotonic phase shift ranging approximately from −218° to +43°, yielding a phase tuning range of over 218°, sufficient for effective beam steering in a phase-gradient metasurface. The reflection magnitude remains relatively stable and high (>0.88) throughout the voltage sweep, ensuring minimal energy loss during phase modulation. Outside this frequency range (i.e., beyond 4.0–4.4 GHz), additional simulations indicate that the phase tunability degrades, which reduces beam-steering effectiveness. This is attributed to the frequency-dependent resonance behavior of the unit structure and the non-linear capacitance-voltage response of the varactor diode.

### 5.3. Electrically Tunable Beam-Steering Metasurface Array Design Simulation

**Figure 12:**
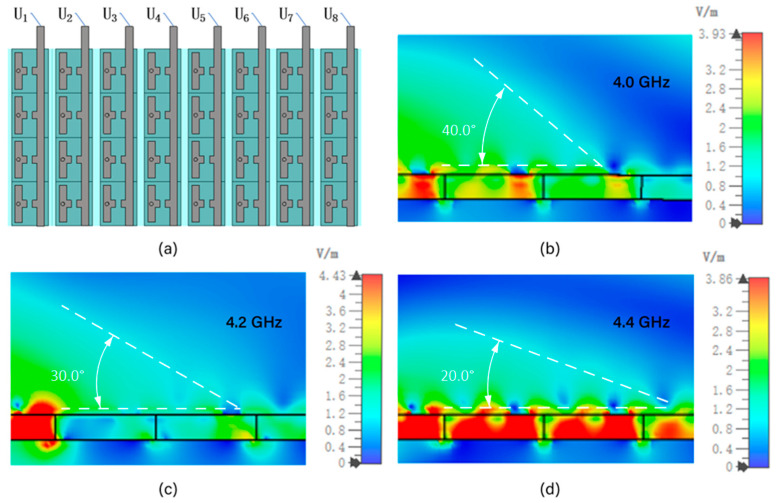
Electrically tunable metasurface structure and simulation results. (**a**) Schematic of the metasurface structure. (**b**) 40° beam steering at 4.0 GHz. (**c**) 30° beam steering at 4.2 GHz. (**d**) 20° beam steering at 4.4 GHz.

After obtaining the simulation results of the metasurface unit, the overall beam steering metasurface was further designed. Based on the metasurface unit designed in the previous Section, a 4 × 8 array was constructed, as shown in Figure 12a. According to Equation (5), the required reflection phases for units 1 to 8 were calculated. However, due to the discontinuous variation in the capacitance of the varactor diode, the resulting reflection phases are also discrete. Therefore, in designing the beam-steering metasurface, phase values close to those calculated by Equation (5) were selected based on the simulation data shown in Figure 11. The corresponding bias voltages were then determined using the voltage–phase relationship also illustrated in Figure 11. According to the working principle shown in Figure 9, the DC voltage generated after rectification is relatively stable, but it is usually continuous and has a large magnitude. In order to provide different voltage levels for the varactor diodes embedded in each metasurface unit, an external resistor voltage divider network is required. This resistor network is composed of a series of resistors connected in series or in parallel. According to the resistance value ratio of the resistors, the rectified DC voltage is proportionally divided into several discrete voltage levels, denoted as U_1_, U_2_, …, U_8_. These voltages after voltage division are respectively arranged through wires or microstrip lines and connected to the bias terminals of the corresponding unit varactor diodes in the metasurface array. For example, the first unit of the metasurface array as the varactor diode, receives the voltage U_1_, the second unit receives the voltage U_2_, and so on. Through circuit design, different unit varactor diodes can simultaneously obtain different bias voltages. This directly leads to a change in the phase of the reflected wave of the unit.

Since the metasurface units along the x-direction were assigned identical capacitance values (corresponding to the same applied bias voltage), a uniform phase response was established in that direction. A linear phase gradient was introduced along the y-direction by applying different voltages to the 8 unit columns, as determined in accordance with the theoretical phase distribution. To simulate the electromagnetic response, a normally incident planar wave was applied from the z-direction using a Floquet port excitation. Periodic boundary conditions were imposed along both x- and y-directions to emulate the array, while lumped elements were used to model the varactor diodes with varying capacitances. The electric field amplitude distribution was recorded using field monitors at a specified observation plane. By controlling the spatial gradient of the capacitance (i.e., the reflected phase) of the metasurface element, the phase modulation in front of the electromagnetic wave can be achieved, thereby changing the direction of the reflected wave and realizing the deflection of the beam.

As shown in Figure 12b–d, the designed beam-steering metasurface successfully achieves beam steering. We simulated the metasurface at 4.0 GHz, 4.2 GHz, and 4.4 GHz, and observed clear beam deflection angles of 40°, 30°, and 20°, respectively. The results demonstrate that clear and distinct beam-steering effects are observed within the 4.0–4.4 GHz range. Beyond this range, the beam-steering performance becomes significantly less effective. This limitation arises from two coupled factors: (i) the non-linear capacitance–voltage relationship of the varactor diode, which results in non-uniform phase tuning steps and discontinuous phase profiles, and (ii) the intrinsic resonance of the unit cell, which confines efficient phase control to a narrow frequency band. Consequently, the deflection angle cannot be arbitrarily tuned outside the optimal band, leading to reduced beam-steering precision. Furthermore, the present metasurface exhibits polarization sensitivity due to the asymmetric unit cell structure, with effective steering occurring for linearly polarized incident waves aligned to the varactor orientation. This polarization dependence may constrain performance in scenarios with varying or unknown polarization states, suggesting that polarization-insensitive unit cell designs should be considered in future work. Nevertheless, integrating the TENG as a voltage source offers a novel and portable approach for electrically tunable metasurfaces, enhancing their practicality for compact beam-steering systems.

## 6. Results and Discussion

An electrically tunable metasurface unit integrating a varactor diode was designed and simulated to realize active phase modulation. The unit structure consists of two parallel metal strips with a varactor diode embedded between them, enabling phase modulation through the adjustment of applied bias voltage. Simulation results presented in Figure 11 demonstrate that under normally incident plane waves, the reflection phase of the unit exhibits a discrete variation with respect to bias voltage, ranging from 0 V to 23 V. This result is consistent with the known non-linear capacitance voltage characteristics of the SMV1430-040LF varactor diode used in this study, whose capacitance varies non-continuously from 1.284 pF to 0.367 pF.

Building on the unit-level results, a 4 × 8 metasurface array composed of individually biased elements was designed to achieve beam steering. However, due to the inherent non-linearity and discreteness of the varactor diode, the available reflection phases are not continuously tunable. To overcome this limitation, we selected phase values from simulation results (Figure 11b–d) that were closest to the target values derived from theory. The corresponding voltages were then applied to the metasurface elements.

To provide a compact and portable voltage source for beam control, a triboelectric nanogenerator was introduced as the energy source. Furthermore, electrostatic simulations of the TENG structure showed that the surface potential difference increases with the separation distance between the friction layers, rising from about 10 V at 0.5 mm to 90 V at 4.5 mm, which supports its ability to generate voltages suitable for varactor tuning. As illustrated in Figure 9, the TENG output was divided through eight resistors (R_1_–R_8_), yielding individual voltages (U_1_–U_8_), which were applied to the respective metasurface units. This integrated simulation model of the TENG–metasurface system represents a meaningful step toward the realization of TENG-driven, field-deployable, and lightweight beam control devices.

These results underscore the promise of this metasurface design in applications, such as reconfigurable intelligent surfaces, tunable antenna arrays, and energy-efficient beam steering systems. As this study is primarily simulation-based, future work will focus on the fabrication and experimental characterization of the complete TENG-metasurface system to validate the proposed concept in real-world conditions.

## 7. Conclusions

In this study, a triboelectric nanogenerator was employed to harvest mechanical energy from human motion and convert it into electrical power, which was then simulated to demonstrate its voltage output. The alternating voltage generated by the TENG was rectified into direct current and applied to a metasurface unit loaded with varactor diodes. Electrically, the metasurface unit can be modeled as a simple series circuit composed of resistance, inductance, and capacitance, where the inductance remains relatively constant, while the resistance and capacitance can be effectively tuned by the applied voltage. This configuration enables precise control of the reflection phase of the metasurface unit. Simulation results show that TENG-generated power successfully modulates the metasurface to deflect a normally incident plane wave at different reflection angles, thereby achieving beam steering. These findings validate the feasibility of integrating energy harvesting and programmable metasurface, offering a promising pathway for developing reconfigurable electromagnetic systems.

It is important to note that the current work is simulation-based, serving as a proof-of-concept study. Future work will focus on the fabrication of the proposed metasurface device and the experimental characterization of its performance, including real-time validation of the energy harvesting and beam steering functionalities. These experimental efforts will be essential to further demonstrate the practical applicability and robustness of the integrated system.

## Figures and Tables

**Table 1:** Experimental instruments and materials.

Item	Model/Name	Manufacturer
Electrometer	Keithley 6514	Keithley Instruments, Inc., Cleveland, OH, USA
Linear Motor	R-LP4	Rigol Technologies Co., Ltd., Beijing, China
Force Gauge	HF-5K	Yueqing Aidebao Instrument Co., Ltd., Wenzhou, China
Polydimethylsiloxane	PDMS	Sichuan Shengjili Industrial Co., Ltd., Chengdu, China
Aluminum	Al	Sichuan Shengjili Industrial Co., Ltd., Chengdu, China
Polymethyl methacrylate	PMMA	Sichuan Shengjili Industrial Co., Ltd., Chengdu, China
Copper	Cu	Ubesure Technology Co., Ltd., Shenzhen, China

**Table 2:** Unit structure dimension parameters.

Parameter	Value	Unit
length 1 (l1)	10	mm
length 2 (l2)	4	mm
length 3 (l3)	2	mm
width 1 (w1)	2	mm
width 2 (w2)	1.35	mm
width 3 (w3)	10	mm

## Data Availability

The data presented in this study is available on request from the corresponding author. The data are not publicly available due to privacy or ethical restrictions.
